# Using Social Media Data to Understand Consumers' Information Needs and Emotions Regarding Cancer: Ontology-Based Data Analysis Study

**DOI:** 10.2196/18767

**Published:** 2020-12-07

**Authors:** Jooyun Lee, Hyeoun-Ae Park, Seul Ki Park, Tae-Min Song

**Affiliations:** 1 College of Nursing Gachon University Incheon Republic of Korea; 2 College of Nursing Seoul National University Seoul Republic of Korea; 3 Department of Health Management Sahmyook University Seoul Republic of Korea

**Keywords:** social media, ontology, cancer, health information needs, cancer information, emotion

## Abstract

**Background:**

Analysis of posts on social media is effective in investigating health information needs for disease management and identifying people’s emotional status related to disease. An ontology is needed for semantic analysis of social media data.

**Objective:**

This study was performed to develop a cancer ontology with terminology containing consumer terms and to analyze social media data to identify health information needs and emotions related to cancer.

**Methods:**

A cancer ontology was developed using social media data, collected with a crawler, from online communities and blogs between January 1, 2014 and June 30, 2017 in South Korea. The relative frequencies of posts containing ontology concepts were counted and compared by cancer type.

**Results:**

The ontology had 9 superclasses, 213 class concepts, and 4061 synonyms. Ontology-driven natural language processing was performed on the text from 754,744 cancer-related posts. Colon, breast, stomach, cervical, lung, liver, pancreatic, and prostate cancer; brain tumors; and leukemia appeared most in these posts. At the superclass level, risk factor was the most frequent, followed by emotions, symptoms, treatments, and dealing with cancer.

**Conclusions:**

Information needs and emotions differed according to cancer type. The observations of this study could be used to provide tailored information to consumers according to cancer type and care process. Attention should be paid to provision of cancer-related information to not only patients but also their families and the general public seeking information on cancer.

## Introduction

Dealing with cancer is both physically and mentally difficult, and patients require information on not only cancer itself but also on how to live with cancer [1-3]. There are time and situation constraints [3] that can hinder fulfillment of these requirements by health care providers such as physicians and nurses. Moreover, such information needs cannot be met by family members owing to their lack of expertise [2].

Health care consumers often use social media to exchange information, share experiences, and seek emotional support. They seek information from social media about diseases, treatments, and statistics to understand the disease and for help in making decisions. They also use social media to relieve anxiety and promote comfort by sharing their experiences and feelings, arising with cancer [1,4,5]. High-quality information is provided by cancer information portals, most of which are operated by government or professional societies. However, these portals are not designed to support sharing of experiences and feelings among patients. Therefore, consumers use social media to interact with each other by writing and reading about their shared experiences and feelings.

People use social media to share opinions, perceptions, concerns, and worries about health conditions [6]. Such posts have proven to be effective in identifying the interests and concerns of health care consumers related to the prevention, diagnosis, treatment, and management of diseases and the emotions related to diseases [7-10]. In recent years, there have been many studies [8,11,12] that extract health-related topics from social media data. The premise of these studies is that the topics posted on social media and their frequencies reflect the extent of consumers’ health information needs [8]. A thorough understanding of these needs would be helpful in providing tailored information to consumers.

Text clustering [8] and machine learning [11,12] have been widely used to extract health-related topics from social media data. Lu et al [7] integrated medical terminology using the Unified Medical Language System to reflect the structure of medical knowledge in text clustering of messages posted by patients with lung cancer, breast cancer, and diabetes on online health communities. They were able to detect health-related topics effectively using this approach. The use of specific ontology for the domain of interest is helpful in the effective identification of relevant topics from social media data.

Although certain cancer-related ontologies, such as those for liver, breast, and gastric cancer, are available, they were developed with professional medical terms for data extraction from or integration with clinical databases [13-15] and are not suitable for analyzing social media data posted in consumer terms. Therefore, it is necessary to develop an ontology with terminology containing consumer terms to analyze social media data posted by consumers.

This study was conducted to develop a cancer ontology with terminology containing consumer terms and to analyze social media data to identify health information needs and emotions related to cancer.

## Methods

The study consisted of 2 stages: (1) development and evaluation of a cancer ontology, and (2) analysis of social media data using the ontology.

### Development and Evaluation of a Cancer Ontology

Ontology development was performed based on previous reports by Noy and McGuinness [16] and Jung et al [17].

First, the domain and scope of the ontology were determined using the following competency questions: (1) What types of cancer are mentioned in posts on social media? (2) Which care delivery processes (eg, prevention, diagnosis, treatment) of cancer in general and specific cancer types are mentioned in social media posts? (3) What cancer-related topics are mentioned in posts for cancer in general and specific cancer types? (4) What emotions, which mean a range of feelings a patient with cancer can experience when dealing with cancer, are mentioned in posts for cancer in general and specific cancer types?

The purpose of the ontology was determined as collecting and analyzing social media data to identify cancer information needs and emotions related to cancer.

Second, existing ontologies on cancer were identified: the Liver Cancer Ontology [13], Breast Cancer Ontology [14], and Gastric Cancer Ontology [15]. Each is limited to a specific type of cancer, and none includes consumer terms. Therefore, a new ontology was developed to include various types of cancer and consumer terms.

An existing ontology on emotion was also identified—the Sentiment Ontology for Social Web [18]. This ontology has top-level classes of emotion as positive, neutral, or negative. These top-level classes were too broad, and the second-level class was too detailed to describe a range of feelings a patient with cancer can experience when dealing with cancer. Therefore, a new ontology reflecting emotions accompanying cancer was deemed necessary.

Third, terms extracted from the 3 existing cancer-related ontologies, the Sentiment Ontology for Social Web, cancer information portals, and social media posts related to cancer were enumerated.

The cancer information portals that were reviewed to extract terms were 2 US websites (the National Cancer Institute [19] and American Cancer Society [20]), 1 UK website (Cancer Research UK [21]), and 2 Korean websites (the National Cancer Information Center [22] and National Health Information Portal by Korea Centers for Disease Control and Prevention [23]).

These portals included information on emotions accompanying cancer and how to manage them. Emotions included across portals were anger, guilt, and depression [19-22]. In addition, the Cancer Research UK website [21] included overwhelmed, denial, anxiety, fear, and sadness; the National Cancer Institute [19] included overwhelmed, denial, anxiety, fear, and sadness, loneliness, hope, and gratitude; and the Korean National Cancer Information Center [22] included overwhelmed, denial, anxiety, hope, and gratitude.

Natural language processing (NLP) was used to extract consumer terms from social media data on cancer. Terms with the same meaning as those extracted from existing cancer-related ontologies and cancer information portals or terms with a new meaning related to cancer were collected as consumer terms. These included (1) heteronyms such as “*jol-eob*” (*graduation* in English, meaning *complete cure*); (2) abbreviations such as “*jaegeom*” (an abbreviation of *jaegeomsa*, meaning *re-test* in English), “*chompa*” (an abbreviation of *cho-eumpa*, meaning *ultrasound* in English), “*holmon*” (an abbreviation of *holeumon*, meaning *hormone* in English); and (3) terms used for herbal medicine and complementary therapies.

Fourth, the classes and their hierarchy and relationships were defined. The collected terms were grouped according to semantic meaning and determined concepts as classes with an independent existence. Hierarchies of the classes were designed based on the relationships of the concepts. The superclass and subclass concepts of the ontology were determined by analyzing the structures of the ontology. A list of synonyms was compiled for each class concept as a terminology presenting the relationship between the concept and synonyms in the ontology.

Nine domains of cancer-related emotions were identified: *overwhelmed, denial, anger, fear and anxiety, sadness and depression, guilt, loneliness, hope,* and *gratitude*. Each domain was defined as a class concept, and a list of synonyms of the class concepts was mapped as a terminology.

Fifth, the structure, correctness, and quality of the ontology were evaluated using the evaluation tool described below and by interviewing 3 domain experts: 2 professors of family medicine and 1 professor of bioinformatics.

The tool consisted of 13 items selected from the studies of Hlomani and Stacey [24] and Kehagias et al [25]. The items were scored on a 5-point scale for structure (size, depth of hierarchy, breadth of hierarchy, balance, overall complexity, and connectivity between concepts), correctness (accuracy, completeness, conciseness, and consistency), and quality (computational efficiency, adaptability, and clarity). Interviews included open-ended questions that allowed experts to recommend revisions of the ontology. The ontology was revised based on the results of the evaluation.

### Analysis of Social Media Data

#### Data Collection and Preparation

The social media data for this study were posts on cancer collected using a crawler from online communities and blogs of 4 social media platforms in South Korea, namely *Naver*, *Daum*, *Tistory*, and *Egloos*, between January 1, 2014 and June 30, 2017.

A total of 302 concepts and synonyms of the *cancer type* superclass were used as keywords for post extraction, and 418 concepts were used as stop keywords. For example, when certain Korean words or morphemes, such as “*agseong*” (meaning *malignant* in English) or “*am*” (meaning *cancer* in English) are combined with other words, the phrase could become a word or morpheme with a completely different meaning, such as “*agseong virus*” (meaning computer virus) or “*an-am*” (name of a district in Seoul). Posts containing 59 advertising keywords (eg, detoxification, antioxidant therapy, and enzyme therapy), suggesting an advertising post were removed.

A total of 754,744 posts were extracted from online communities and blog sites. When categorized by source into blogs and online communities, 442,669 (58.7%) were blog posts. Of the 754,744 posts, 234,118 were from 2014; 235,509 were from 2015; 200,553 were from 2016; and 84,564 were from the first half of 2017. Most of the posts (737,575; 97.7%) were from *Naver* and *Daum*, the 2 major social media platforms in South Korea.

Next, ontology-based NLP was performed on the posts to extract class concepts.

The data collection and NLP were carried out in collaboration with a Korean telecommunications company (Smart Insight). During NLP, identifying information (such as name, phone number, and account) was removed, and masked data were delivered to the research team.

#### Frequency Analysis of Posts

The unit of analysis was the post, and the frequencies of posts containing single specific class concepts were counted.

First, the relative frequencies of posts containing specific cancer types were counted and compared with the national cancer statistics of Korea. Top-ranked cancer types in social media posts were selected for further analyses. Second, the relative frequencies of posts containing superclass concepts were counted and compared by cancer type. Finally, the frequencies of posts containing end-node class concepts were counted and organized by cancer type.

None of the posts used in this study had any identifying information. The study was approved by the Institutional Review Board of Seoul National University (No. 1802/001-006).

## Results

### Development and Evaluation of the Ontology

Based on the existing cancer-related ontologies and cancer information portals, 10 superclasses were identified: *cancer type, prevention, diagnosis, treatment, prognosis* (including *recurrence* and *cure*), *risk factor, symptom, side effect, dealing with cancer,* and *emotion*. The ontology consisted of concepts that represented care delivery processes and patient outcomes, such as *prevention, diagnosis, treatment,* and *prognosis*, and another set of concepts that represented how consumers managed, felt, perceived, and acted in their personal lives, such as *risk factor, symptom, side effect, dealing with cancer,* and *emotion*.

The average scores by the 3 experts of 13 items designed to evaluate the structure, correctness, and quality of the ontology ranged from 4.33 to 5 on the 5-point scale. The score for correctness was the highest (mean 4.83), followed by structure (mean 4.67) and quality (mean 4.67). A suggestion made by one of the experts was to combine the superclass of *side effect* with that of *symptom*, moving cancer symptoms and treatment side effects into subclasses of the *symptom* superclass, because it is difficult to distinguish side effects from symptoms without context. Another suggestion was to add the national cancer support system as a subclass of the *dealing with cancer* superclass. The ontology was revised to reflect the comments made by the experts.

**Figure 1:**
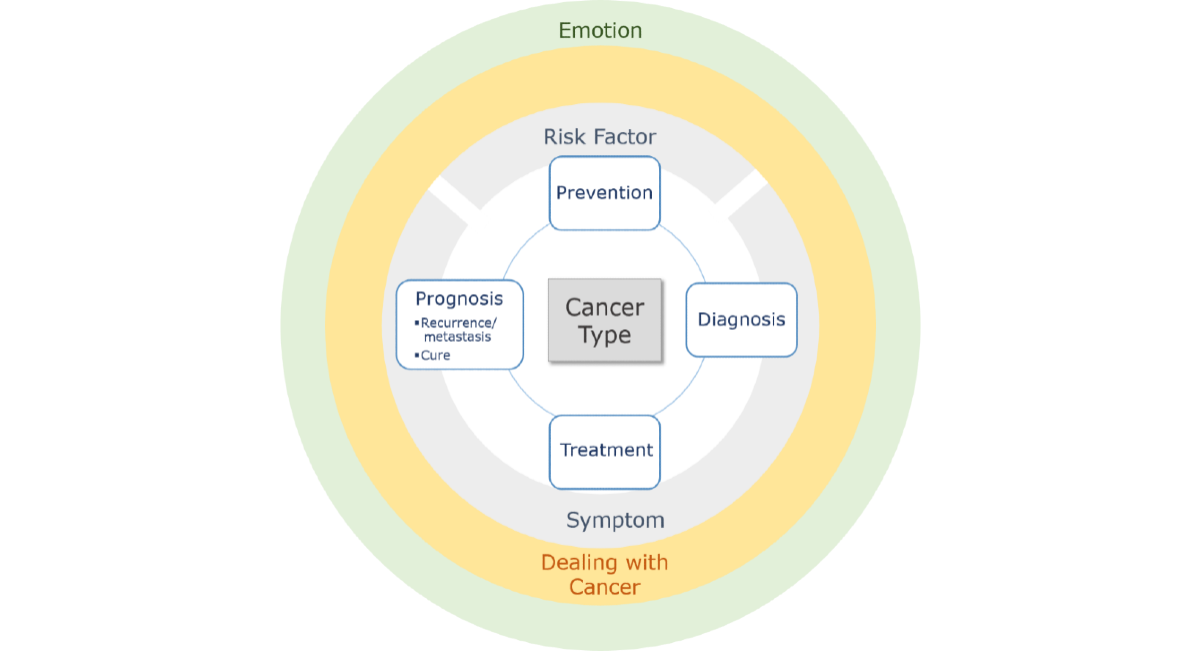
Cancer-related ontology superclasses.

The revised ontology had 9 superclasses, 213 class concepts, and 4061 synonyms. It had 3 to 4 levels of hierarchy, with 36 first-level subclasses and 41 second-level subclasses (Figure 1).

### Analysis of Social Media Data

#### Comparison of Top-Ranked Cancer Types

**Figure 2:**
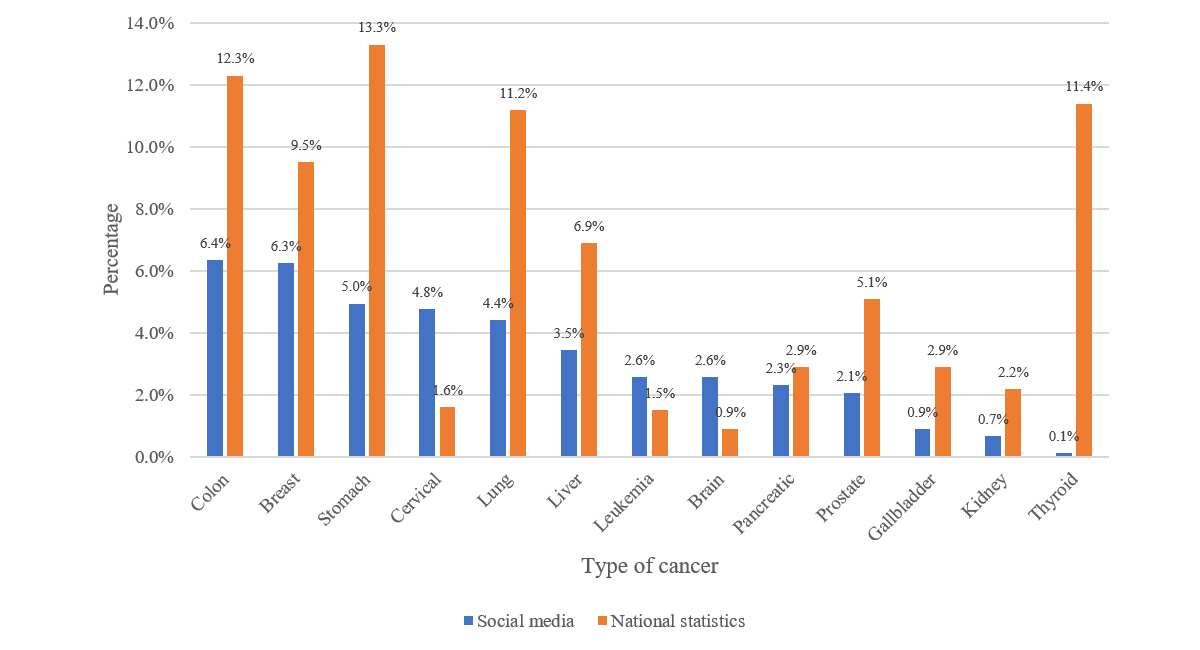
Comparison of top-ranked cancer types in social media posts and national cancer statistics.

Figure 2 presents the top-ranked cancer types mentioned on social media compared with national cancer statistics. Colon cancer (47,940/754,744, 6.4%) was the most frequently mentioned on social media, followed by breast cancer (47,235/754,744, 6.3%) and stomach cancer (37,378/754,744, 5.0%). The 4 highest-ranked cancers according to the national cancer statistics were stomach, colon, thyroid, and lung cancer.

Colon, breast, stomach, lung, liver, prostate, and pancreatic cancer were within the top 10 rankings in both social media data and the national cancer statistics. Cervical cancer, leukemia, and brain tumors, which ranked within the top 10 cancer types in the social media data, were not included in the top 10 cancer types in the national cancer statistics. Thyroid cancer ranked within top 3 in the national cancer statistics but was not included in the top-ranked cancer types on social media.

#### Frequency of Posts Containing Superclass-Level Concepts

In terms of the frequency of posts at the superclass level, *risk factor* was the most frequent, appearing in 42.5% (320,568/754,744) of the posts, followed by *emotion* in 33.8% (254,920/754,744), *symptom* in 32.2% (243,010/754,744), *treatment* in 30.2% (227,942/754,744), *dealing with cancer* in 29.4% (221,996/754,744), *diagnosis* in 23.7% (178,498/754,744), *prevention* in 14.4% (108,408/754,744), and *prognosis* in 9.4% of the posts (70,583/754,744).

##### Risk Factor Superclass

**Figure 3:**
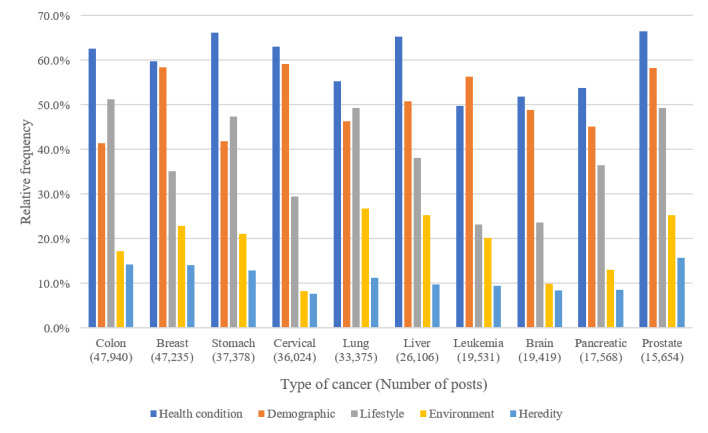
Relative frequencies of the risk factor superclass by top 10 cancer types in social media posts.

Figure 3 shows the relative frequencies of posts containing *risk factor* superclass concepts for 10 specific cancer types. *Health condition-related risk factor* class concepts (such as related disease and health status) were the most common in most cancer types, followed by *demographic*, *lifestyle* (such as diet and exercise), *environmental*, and *hereditary risk factor* concepts.

*Demographic risk factor* class concepts appeared more frequently in posts on breast cancer, cervical cancer, leukemia, and prostate cancer than in those on other types of cancer. *Lifestyle-related risk factor* class concepts were more frequent in posts on colon, stomach, and lung cancer than in those on other cancer types. *Environmental risk factor* class concepts were more frequent in posts mentioning lung, liver, and prostate cancer than in those on other cancer types.

##### Care Process of Cancer: Prevention, Diagnosis, Treatment, Recurrence, and Cure Superclasses

**Figure 4:**
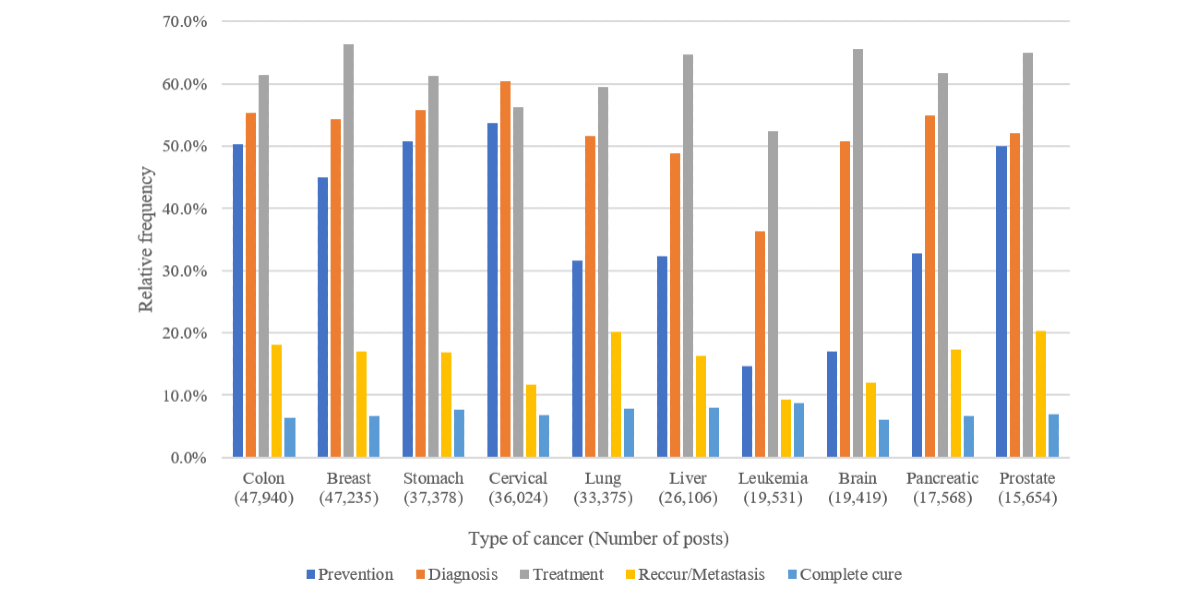
Relative frequencies of superclass concepts related to the care process of cancer by top 10 cancer types in social media posts.

Figure 4 shows the relative frequencies of posts containing superclass concepts related to the cancer care process by 10 specific cancer types. Posts containing *treatment* class concepts were the most common, followed by those related to *diagnosis, prevention*, *recurrence,* and *cure* class concepts for most cancer types, except for cervical cancer.

*Prevention* class concepts were more frequent in posts on cervical, stomach, colon, and prostate cancer and less frequent in posts on brain tumors and leukemia. The *diagnosis* class was dominant in cervical cancer posts, appearing more frequently than the *treatment* class. The *recurrence* class was the least frequently mentioned in leukemia posts, in which the *cure* class was common.

##### Symptom Superclass

**Figure 5:**
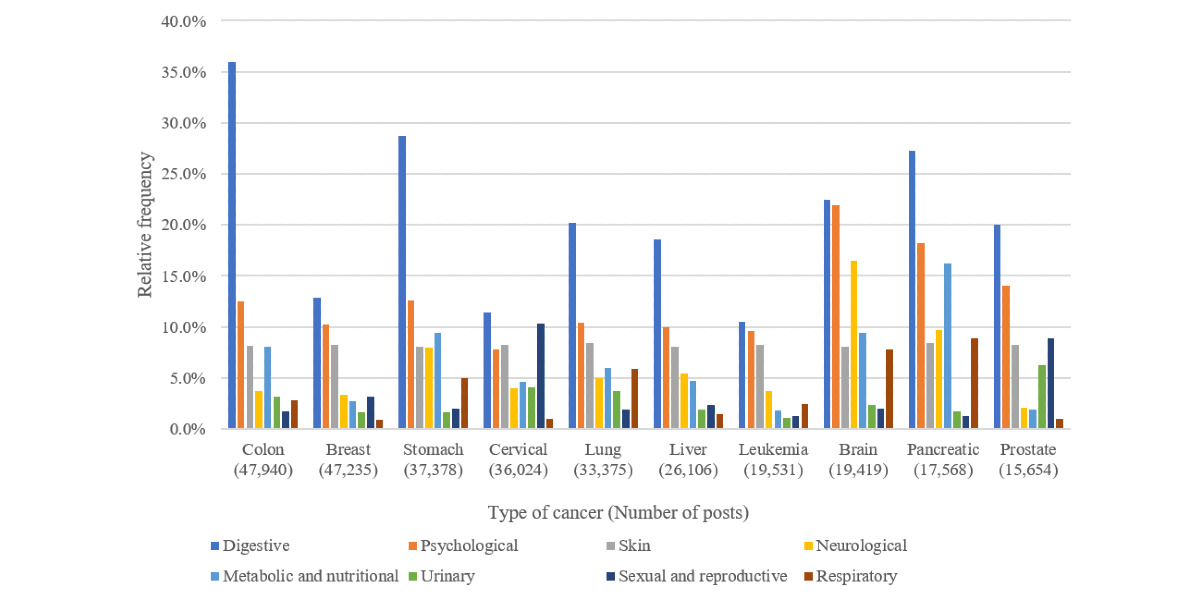
Relative frequencies of the symptom superclass by top 10 cancer types in social media posts.

Figure 5 shows the relative frequencies of posts containing *symptom* superclass concepts by 10 specific cancer types. *Digestive symptom* class concepts were the most common in posts on all cancer types, followed by *psychological symptom* concepts.

*Digestive symptom* class concepts were predominant in posts related to cancers of the digestive system, such as colon, stomach, and pancreatic cancer. *Psychological and neurological symptom* class concepts appeared more frequently in posts on brain tumors than in those on other cancers. *Metabolic symptom* class concepts were frequent in posts on pancreatic cancer, and *sexual and reproductive symptom* class concepts were frequent in posts on cervical and prostate cancer.

##### Dealing With Cancer Superclass

**Figure 6:**
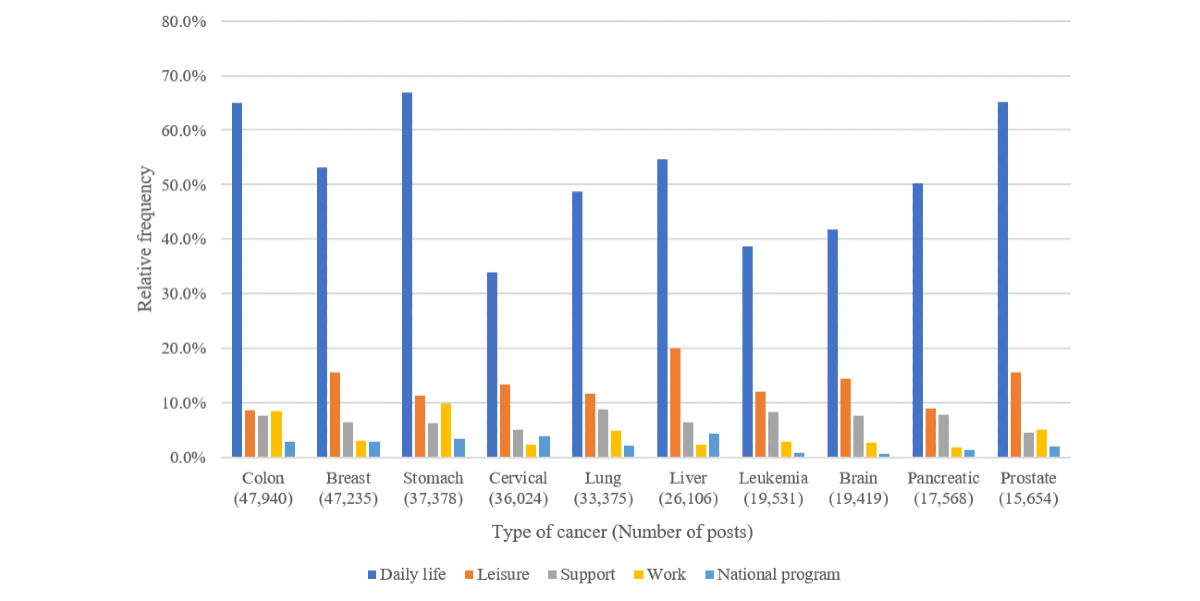
Relative frequencies of the dealing with cancer superclass by top 10 cancer types in social media posts.

Figure 6 shows the relative frequencies of posts containing *dealing with cancer* superclass concepts by 10 specific cancer types.

*Daily life* class (involving diet and exercise) concepts were predominant in posts on the 10 cancer types. The most noticeable class concept in posts on *daily life* was *diet*. *Leisure* class concepts (involving sex life, travel, and driving) were more common in posts on liver and breast cancer.

##### Emotion Superclass

**Figure 7:**
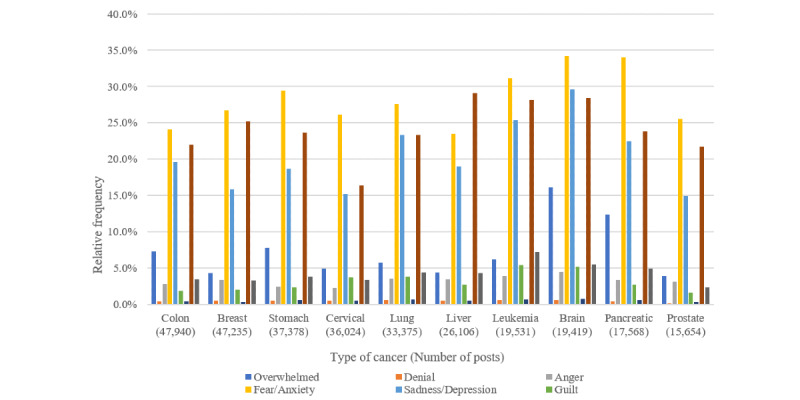
Relative frequencies of the emotion superclass by top 10 cancer types in social media posts.

Figure 7 shows the relative frequencies of posts containing *emotion* superclass concepts by 10 specific cancer types. *Fear/anxiety* class concepts were the most common, followed by *hope* and *sadness/depression* concepts, in posts on all cancer types except for liver cancer and brain tumors.

*Hope* class concepts were more frequently mentioned than *fear/anxiety* class concepts in posts on liver cancer. *Overwhelmed* class concepts were more frequent in posts on brain tumors and pancreatic cancer than in those on other cancers. *Gratitude* and *guilt* class concepts were more frequently mentioned in posts on leukemia than in those on other cancers.

#### Frequencies of Posts Containing Class-Level Concepts by Cancer Type

**Figure 8:**
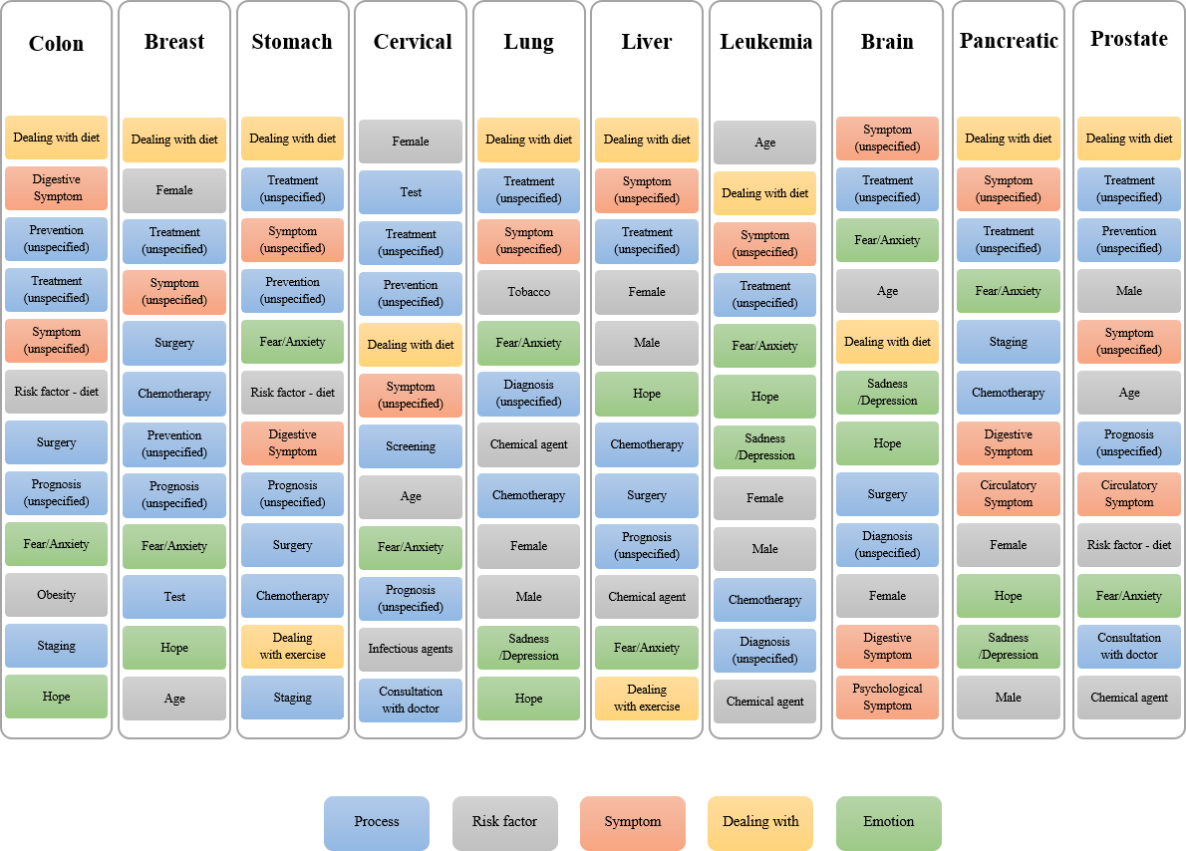
Top ranked end-node class concepts by cancer type.

Figure 8 shows the top-ranked end-node class concepts in order of relative frequency by cancer type.

The *dealing with diet* class was ranked first in posts related to 8 cancer types. The dominant concepts and terms appearing in posts about *dealing with diet* were food, protein, fruit, vitamin, meal, vegetable, and nutrition.

Class concepts related to the care process of cancer were frequently highly ranked; the *unspecified treatment* class was highly ranked for all types of cancer. The *surgery* class was ranked within the top 10 class concepts in posts on colon, breast, stomach, and liver cancer, and brain tumors. The *chemotherapy* class was ranked within the top 10 class concepts in posts on breast, stomach, lung, liver, and pancreatic cancer, and leukemia.

Several risk factor–related class concepts ranked in the top 10 class concepts, but the order differed by cancer type. *Gender* and *age* classes frequently appeared in posts on breast, cervical, prostate cancer, and leukemia. The *dietary risk factor* class frequently appeared in posts on colon, stomach, and prostate cancer, and *obesity* class frequently appeared in posts on colon cancer. The *chemical risk factor* class frequently appeared in posts on lung, liver, and prostate cancer, and leukemia. The *infectious agent* class (such as viruses) frequently appeared in posts on cervical cancer.

There were no posts containing *support group/support community* class concepts in posts on thyroid cancer, but these concepts appeared in posts on all of the other top 10 cancers.

## Discussion

### Principal Findings

In this study, an ontology containing consumer terms was developed to collect and analyze social media data to identify health information needs and emotions related to cancer. The ontology has the following characteristics.

First, compared to other cancer ontologies, this ontology covers more cancer types and comprehensive topics, including the care process describing the interactions of health care providers and patients, risk factors that consumers have, emotions that consumers feel related to cancer, symptoms that consumers perceive, and the lifestyles that consumers lead.

Second, the ontology has a terminology component that presents consumer terms to analyze social media posts about cancer, including synonyms, heteronyms, and abbreviated expressions.

A total of 754,744 cancer-related posts on social media were collected. The 6-month collection of 2017 posts was less than half that of the full-year collection of the previous year. Monthly analysis showed cancer-related social media posts were more frequent in the second half of the year than in the first half of the year. In particular, the number of posts in February 2014 was almost twice the monthly average, which was the same as the search results of Google Trends. According to Google Trends, the cancer diagnosis and death from cancer of 2 Korean celebrities had been announced at this time.

Social media data were analyzed using NLP with ontology concepts and terms to identify consumers’ information needs. The frequencies of topics in social media data may indirectly reflect consumers’ information needs, as they frequently post on topics in which they are interested or about which they have concerns [8].

Thyroid cancer, which ranked in the top 3 in the national cancer statistics, was not included in the top-ranked cancer types on social media. This finding is similar to that of Buis and Whitten [26], showing that the information needs related to cancers with a low survival rate are higher than those for cancers with a high survival rate. The 5-year survival rate for thyroid cancer in Korea was about 100% in 2013-2017, compared to 70.4% for all types of cancer [22].

Frequency analysis by superclass revealed the highest frequency of posts on social media related to *risk factor* (320,568/754,744, 42.47%), followed by *emotion* (254,920/754,744, 33.78%), *symptom* (243,010/754,744, 32.20%), and *treatment* (227,942/754,744, 30.20%). These findings can be compared to those of 2 previous studies: Lu et al [7] clustered posts of patients with lung and breast cancer and extracted symptoms, examinations, and treatments (drugs, procedures) as dominant topics. Cho et al [1] performed a qualitative content analysis of Q & A posts of patients with breast cancer, and extracted treatment, physical condition, and lifestyle/self-care as dominant topics. They also found that 75% of the information requests included expressions of emotion [1]. Although these previous studies [1,7] focused mainly on the stages after diagnosis, such as treatment, examinations, and physical condition, our results revealed that the majority of consumers’ information needs involved risk factors. Also, previous studies [1,7] used data collected from cancer community posts by patients with specific cancer types, whereas this study used data collected from social media posts by the general public. Social media data may include concerns of the public about cancer risk factors. In addition, as the ontology developed in this study covered comprehensive topics, including risk factors and emotions, this study had a more diverse focus than those of previous studies [1,7].

Consistent with the findings of the 2 previous studies [1,9], the most common emotions related to the top 10 cancers in this study were *fear/anxiety, hope*, and *sadness/depression*. Cho et al [1] reported that anxiety/worry, gratitude, fear, and sadness were frequent in posts by Korean women with breast cancer, and Freedman et al [9] reported that fears, anxiety, denial, and depression were frequent emotions cited on treatment in posts by patients with breast cancer. However, among positive emotions, gratitude was included in the top 3 in the study by Cho et al [1], whereas hope was ranked in the top 3 in our study. The question and answer board in Cho et al's study [1] included more posts on gratitude toward health care providers, in contrast to social media posts in this study that often included posts on hope for a cure by patients and their families.

Frequency analysis by the end-node class level indicated that *dealing with diet* ranked among the top-class concepts for most types of cancer. This finding was consistent with those of previous information-need studies [1,27] in Korean patients with breast cancer. Cho et al [1] reported that patients asked many questions related to diet, and Kim and Hur [27] reported high information-need scores for diet. These findings reflect the importance of diet in disease management that is perceived by the Korean population. Concepts and terms such as food, protein, fruit, vitamin, meal, vegetable, and nutrition were top ranked in the posts collected in this study, demonstrating consumers’ information needs related to a healthy diet.

This study also compared the frequencies of superclass concepts and end-node class concepts by 10 types of cancer.

Regarding the care process of cancer, *treatment* was the most frequently mentioned for all types of cancer, except cervical cancer. *Diagnosis* and *prevention* appeared more frequently in posts on cervical cancer. In Korea, the national cancer screening program for cervical cancer is recommended from 20 years of age. Whereas breast and gastric cancer screenings are recommended from 40 years of age, and colorectal cancer screening is recommended from 50 years of age. As the opportunity to become interested in screening for cervical cancer comes earlier than for other cancers, the active use of social media by young women may have resulted in greater numbers of posts related to diagnosis and prevention.

The frequencies of risk factor–related class concepts, especially *diet, chemical, tobacco, obesity,* and *infectious agent*, differed between cancer types. Previous research findings have indicated that differences in post frequency on cancer topics reflect the different information needs for each cancer type [7,8]. Therefore, tailored information on risk factors should be provided according to consumers’ needs by cancer type. In addition, tailored information on cancer can be provided according to the consumers’ specific risk factors.

Posts on leukemia contained a higher rate of feelings of *guilt*. According to the National Cancer Information Center [22] data, leukemia has a higher incidence in children than those of other types of cancer. In many cases, posts related to leukemia were likely written by the parents of patients rather than by the patients themselves. This finding suggests that emotional management is necessary for not only patients with cancer but also their family members and friends.

Based on these findings, we suggest what information should be provided and how it can be provided. These suggestions would aid information providers, namely clinicians and portals operated by government or professional societies, to improve care for patients with cancer by providing relevant information based on consumers’ information needs.

First, it is necessary to ensure that sufficient information on risk factors is provided to the public. Not only do information needs increase after cancer is diagnosed, but they are also high for risk factor management. Thus, information on risk factors should be provided depending on the concerns of consumers.

Second, the high consumers’ information needs on healthy diets were noteworthy. Qualified information on diet should be provided to patients with cancer. Collaboration with a nutritionist would be effective in providing tailored nutritional information for each cancer type according to the needs of patients.

Third, in general, most information portals provide information through the same organization and the same flow of information for all types of cancer. Tailored information can be provided according to cancer type and the characteristics of the consumer, such as age, gender, and risk exposure. It is possible to make it easier to access the information that consumers want using keyword visualization or navigation. Different navigation routes could be applied according to cancer type. In addition, applying different types of visualization could improve the convenience of consumers before or after the cancer diagnosis.

Finally, providing information and emotional support are not separate but, instead, coexist. It is necessary to provide reliable information and management for emotional care so that people do not rely on only family and caregivers for emotional support. One possible approach is to combine the functions of online support groups with information portals to provide emotional support.

### Limitations

This study had some limitations. Only the top 10 cancer types were analyzed. Future studies should analyze data on other types of cancer. The social media posts were made by not only patients with cancer but also caregivers and the general public. However, these populations could not be distinguished because identifying information of the consumers was not collected. Further research is needed to collect social media data with an identification algorithm to distinguish the status of the authors of the posts and to provide tailored information.

### Conclusion

This study was performed to develop a cancer ontology with terminology containing consumer terms to collect and analyze social media data. The ontology consisted of 9 superclasses (*cancer type, prevention, diagnosis, treatment, prognosis, risk factor, symptom, dealing with cancer,* and *emotion*), 213 classes, and 4061 synonyms with consumer-generated terms. It used 9 emotional classes (*overwhelmed, denial, anger, fear and anxiety, sadness and depression, guilt, loneliness, hope,* and *gratitude*) to investigate emotional status in the social media data on cancer.

This ontology, containing comprehensive cancer-related topics, enabled identification and comparison of consumer interests and concerns about risk factors, dealing with cancer, and emotions as well as the care process in social media data. The results of this study showed that information needs and emotions differ according to cancer type. These observations could be used to provide tailored information to consumers according to the cancer type and care process. Care for patients with cancer can be improved by providing relevant information based on consumers' information needs.

## Abbreviations

NLP: natural language processing
